# A qualitative study on the acceptability and preference of three types of long-lasting insecticide-treated bed nets in Solomon Islands: implications for malaria elimination

**DOI:** 10.1186/1475-2875-8-119

**Published:** 2009-06-04

**Authors:** Jo-An Atkinson, Albino Bobogare, Lisa Fitzgerald, Leonard Boaz, Bridget Appleyard, Hilson Toaliu, Andrew Vallely

**Affiliations:** 1 Pacific Malaria Initiative Support Centre, School of Population Health (SPH), University of Queensland, Brisbane, Australia; 2 Vector Borne Disease Control Programme, Ministry of Health, Honiara, Solomon Islands; 3 Australian Centre for International and Tropical Health, a joint programme of Queensland Institute of Medical Research (QIMR) and SPH, UQ, Brisbane, Australia; 4 Save the Children, Port Vila, Vanuatu

## Abstract

**Background:**

In March 2008, the Solomon Islands and Vanuatu governments raised the goal of their National Malaria Programmes from control to elimination. Vector control measures, such as indoor residual spraying (IRS) and long-lasting insecticidal bed nets (LLINs) are key integral components of this programme. Compliance with these interventions is dependent on their acceptability and on the socio-cultural context of the local population. These factors need to be investigated locally prior to programme implementation.

**Method:**

Twelve focus group discussions (FGDs) were carried out in Malaita and Temotu Provinces, Solomon Islands in 2008. These discussions explored user perceptions of acceptability and preference for three brands of long-lasting insecticide-treated bed nets (LLINs) and identified a number of barriers to their proper and consistent use.

**Results:**

Mosquito nuisance and perceived threat of malaria were the main determinants of bed net use. Knowledge of malaria and the means to prevent it were not sufficient to guarantee compliance with LLIN use. Factors such as climate, work and evening social activities impact on the use of bed nets, particularly in men. LLIN acceptability plays a varying role in compliance with their use in villages involved in this study. Participants in areas of reported high and year round mosquito nuisance and perceived threat of malaria reported LLIN use regardless of any reported unfavourable characteristics. Those in areas of low or seasonal mosquito nuisance were more likely to describe the unfavourable characteristics of LLINs as reasons for their intermittent or non-compliance. The main criterion for LLIN brand acceptability was effectiveness in preventing mosquito bites and malaria. Discussions highlighted considerable confusion around LLIN care and washing which may be impacting on their effectiveness and reducing their acceptability in Solomon Islands.

**Conclusion:**

Providing LLINs that are acceptable will be more important for improving compliance in areas of low or seasonal mosquito nuisance and malaria transmission. The implications of these findings on malaria elimination in Solomon Islands are discussed.

## Background

Solomon Islands (SI) and Vanuatu have the highest documented incidence of malaria in the Asia/Pacific region with an annual incidence rate of 130.9 per 1,000 population in SI and 24.3 per 1,000 population in Vanuatu in 2007 [1]. Malaria causes significant human suffering and impacts on social and economic development. The economic impact of malaria extends further than the direct cost of case management. Indirectly, malaria effects external investments into endemic countries and tourism, it causes a loss of productivity due to work absenteeism and impacts return on education investment [2]. In Solomon Islands, the cost to government of school absenteeism as a result of *Plasmodium falciparum *malaria was estimated to be US$108,966 in 1990, which was a large proportion of the education investment for that year [3]. Loss of education investment and other direct and indirect costs of malaria can have a devastating impact on a small island nation such as Solomon Islands. Countries with intensive malaria have been shown to have income levels of one third less than countries without malaria, however, those countries that have eliminated malaria have usually had substantially higher economic growth in the subsequent five years [4].

In March 2008, Solomon Islands and Vanuatu governments raised the goal of their National Malaria Programmes (NMPs) from control to elimination, with one province in each country targeted for early success by 2014 (Temotu Province, Solomon Islands; Tafea Province, Vanuatu). In addition to this important commitment, both countries are members of the recently formed Malaria Elimination Group (MEG) and the Asia Pacific Malaria Elimination Network (APMEN).

Vector control measures, such as indoor residual spraying (IRS), long-lasting insecticidal bed nets (LLINs) and, in some situations, source reduction, are key integral components of the expanded malaria control and elimination programme in Solomon Islands. Insecticide-treated bed nets have long been advocated for personal protection against vector-borne diseases including malaria. Treated bed nets can have a powerful impact on mosquito density and sporozoite rate. Trials in Tanzania, Western Kenya, and central China have shown that bed nets can have a mass effect in reducing vector populations when a high percentage of community coverage is achieved [5-7]. However, compliance with bed net use can be dependent on attitudes (towards malaria and acceptability of bed nets) and the socio-cultural context of the population.

Acceptability of long-lasting insecticidal nets (LLINs) by individuals and communities in South East Asia and the Western Pacific can be affected by a number of factors. These include perceptions and misconceptions of disease causation and risk; the perceived value, safety and effectiveness of the nets; socio-economic factors; gender issues; previous experience and the inconvenience that they may impose on individual lifestyles and household routines [8-10]. Specifically, some barriers identified to the proper and consistent use of bed nets relating to their acceptability include; that multiple nets can be impractical to mount in small village houses; they are inconvenient and stuffy during the hot season; that they are incompatible with sleeping around a fire in the cold season; that they hinder night time activities and mobility; fear of side effects of the chemical; a lack of mosquito biting nuisance; lack of fear for malaria; and non-belief in the benefit of disease prevention [8-10]. In addition, the use of bed nets may require changes of established sleeping patterns and a reorganisation of domestic space [11].

Characteristics of the various brands of LLINs such as durability, texture, mesh size, net size, shape and colour and insecticide effectiveness may also affect bed net acceptability and use, and needs to be investigated locally prior to programme implementation. Additionally, preference for cost and delivery methods for bed nets can have a considerable impact on their distribution and uptake [12,13]. A challenge for malaria control and elimination in SI and elsewhere is not only achieving sufficient LLIN coverage in communities but identifying and addressing the behavioural factors that impact on their use. Providing populations with LLINs they find acceptable could improve user compliance and may contribute to the overall success of the programme.

A previous study in SI has investigated the attitudes of bed net users and their compliance in using bed nets. Among 124 participants from nine villages in central Malaita, 73% of respondents identified malaria as a major problem, and 85% ranked malaria as the most common disease of children. Yet only 52% of households were classified as high compliers of bed net use (year round use), with the remaining 46% rated as low compliers [9]. Mosquito nuisance was identified as the main determinant of bed net use. Those that used bed nets for protection against malaria were more likely to use bed nets than those who used them for mosquito nuisance alone when mosquito density was low. Hot weather was found to be the primary deterrent for bed net use [9].

Manufacturers of LLINs have attempted to address the problems associated with bed net use by rural populations living in hot climates by using durable materials and increasing mesh size to allow increased ventilation. Anecdotal evidence collected in Solomon Islands by the Vector Borne Disease Control Programme (VBDCP), Ministry of Health (MoH) suggests that Olyset^® ^(Sumitomo Chemicals, Osaka, Japan) LLINs are less popular than PermaNet^® ^2.0 (Vestergaard-Frandsen, Copenhagen, Denmark) LLINs because the former have a stiff texture, wrinkle after washing (so that they become shortened and difficult to fold under bedding) and have a large mesh size that allows mosquitoes to penetrate the net to feed. A similar preference for PermaNet^® ^was found by Das *et al *in India and Nepal where both nets were said to be acceptable, but PermaNet^® ^nets were preferable [14]. These findings are in contrast to the experience in Vanuatu, Africa and Asia, where Olyset^® ^nets have been widely accepted. It is unclear why there is a disparity in the acceptability of Olyset^® ^between the Solomon Islands and other regions and a lack of literature investigating the factors that affect LLIN acceptability in the SW Pacific.

It is imperative that the issue of LLIN acceptability and preference be resolved prior to their procurement and distribution as part of the expanded, intensified response to malaria in Solomon Islands. To address this important operational priority a third type of LLIN, DuraNet^® ^(Clarke Mosquito Control, USA) was included in this research, which has interim approval by the World Health Organization Pesticide Evaluation Scheme (WHOPES) and which shares some of the texture and user characteristics of both PermaNet^® ^and Olyset^® ^(Table 1).

**Table 1:** Characteristics of long-lasting insecticide-treated bed nets (LLINs) used in the study

BRAND	MATERIAL	INSECTICIDE	MESH SIZE	FIBER THICKNESS
Olyset^®^ (Sumitomo Chemical Company, Japan)	Polyethylene	1,000 mg/m^2 ^permethrin	4 × 4 mm	150 denier

PermaNet^® ^2.0 (Vestergaard-Frandsen, Denmark)	Polyester	55 mg/m^2 ^deltamethrin	1.5 × 1.5 mm	100 denier

DuraNet^®^ (Clarke Mosquito Control, USA)	Polyethylene	261 mg/m^2 ^alphacypermethrin	2 × 2.5 mm	145 denier

This paper reports the findings of a qualitative study that aimed to investigate user perceptions of acceptability and preference for different types of LLINs in SI and to identify barriers to their proper and consistent use. The findings were used to inform and refine survey parameters for a larger randomized controlled acceptability and preference trial. This paper also discusses the implications of these findings for the malaria elimination programme in Solomon Islands.

## Methods

### Study area and target population

**Figure 1:**
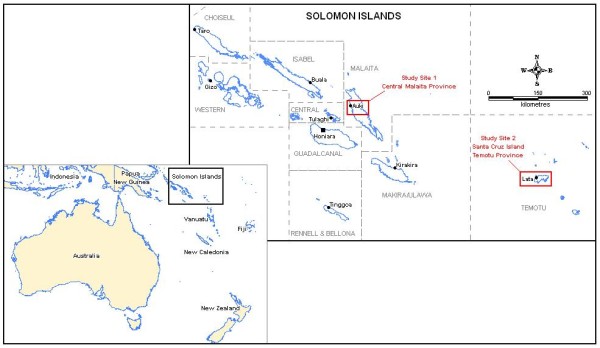
Map of the location of study sites.

This study was conducted from April to June 2008 when malaria transmission in the study areas is generally lower. This was thought to reduce overestimation of the frequency of bed net use. The study sites were purposefully selected to capture the views of those living in areas identified by policy makers as having considerable importance. Temotu is a remote Province and has been selected for malaria elimination. Malaita Province has one of the highest incidences of malaria in the Solomon Islands and will be targeted for intensified control. Due to differences in mosquito density and the potential for differences in attitudes, one coastal and one inland village were selected from each of these Provinces, namely Gwaunaru & Busurata (in central and west Malaita) and Otomongi & Noipe (on the main Island of Santa Cruz, Temotu) (Figure 1).

Housing in the study villages is predominately traditional with bamboo walls and thatched rooves. Sleeping mats made of coconut leaves are used by most of the rural population. Many of the villagers are subsistence farmers and fishermen and income is derived from the sale of this produce in Auki and Honiara. Women are mainly involved in agriculture and domestic duties. The majority population of Solomon Islands is ethnically Melanesian with a small percentage of Polynesian inhabitants on the outer islands of Temotu Province. Melanesian languages are spoken in many Provinces and while English is the official language, the lingua franca is Solomons Pidgin.

A convenience method was used to select the study population. With counsel from a team of in-country public health and research officers, two 'typical' villages were selected from each Province; one inland, one coastal. Village leaders were responsible for recruiting participants, with support from research officers (to encourage the capturing of a variety of views in the community).

### Procedure

**Figure 2:**
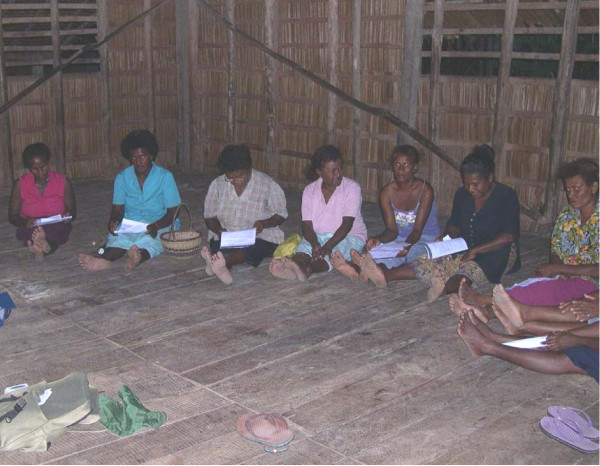
A focus group discussion with primary caregivers underway in Temotu Province, Solomon Islands.

Twelve focus group discussions (FGD) were carried out by trained locally contracted research officers. These research officers were all male. They were provided training in conducting FGDs, data management and study logistics, and were supported by an experienced social scientist based at the School of Population Health (SPH), University of Queensland. Separate FGDs were conducted with male household heads, female primary caregivers and youth (mixed gender) in order to facilitate open, constructive dialogue (Figure 2).

**Figure 3:**
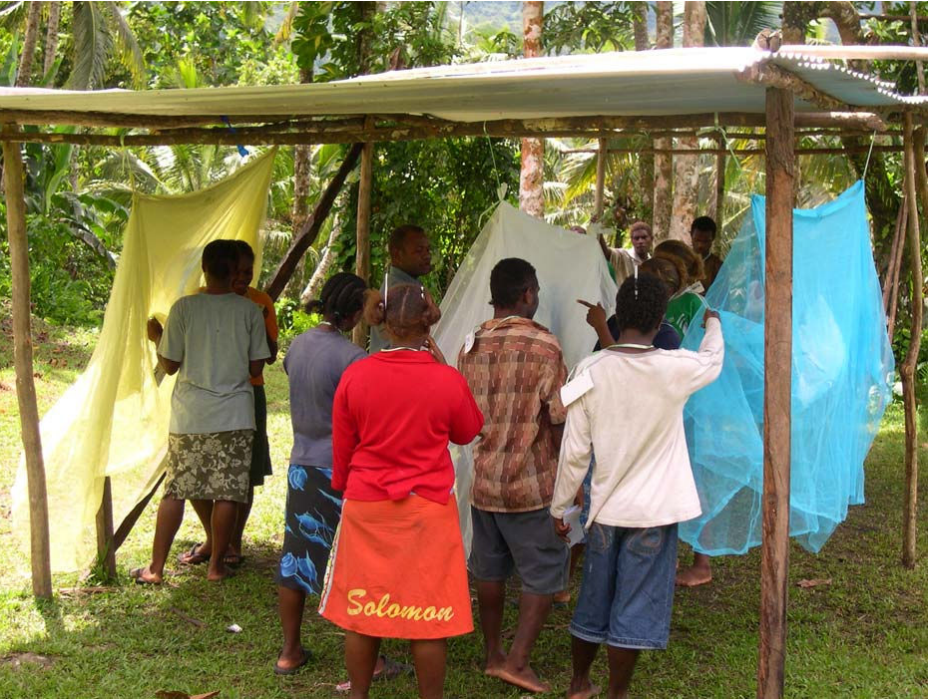
Youth group examining different brands of LLINs during a focus group discussion in Malaita Province, Solomon Islands.

A structured interview guide was used to direct discussions, which focused on knowledge of the purpose of bed nets (including related rumours or misconceptions of LLINs); factors influencing compliance with their use; perceived problematic and favourable characteristics of LLINs; and acceptability and preference of the three brands of LLINs presented (Olyset^®^, PermaNet^® ^and DuraNet^®^). All FGDs were conducted in Solomon Island Pidgin and were digitally recorded. Midway through the discussion, the three brands of LLINs being considered for their acceptability and preference were hung to allow participants time to inspect their qualities (Figure 3). Olyset^® ^and PermaNet^® ^nets have been distributed in these Provinces in the past, but DuraNet^® ^has not been trialled in Solomon Islands.

### Data analysis

Digital recordings of the FGDs were directly transcribed and translated from Pidgin to English by the local research officers. Data was coded around the main topics of the interview guide and entered into NVivo 8 software where key themes were drawn out of the interviews and subjected to thematic analysis by the lead investigators. Thematic analysis is a flexible approach to identifying, analysing and reporting patterns within data and is compatible with 'both essentialist (realist) and constructionist paradigms' [15]. It can be ideal for both reflecting reality and for deconstructing the facade of 'reality.' In the analysis of this study, areas of consensus and divergence were identified and a 'realist method' was used to reflect participants' realities, experiences and meanings. The researchers selected this method of data analysis as it is reportedly advantageous for working within a participatory paradigm and suited to informing policy development [15].

### Ethical aspects

This research was approved by the National Health Research Ethics Committee, Solomon Islands and the Medical Research Ethics Committee, University of Queensland, Australia. Individual informed consent (written or witnessed thumb print) was obtained from all participants prior to the FGDs following a verbal and written explanation of study aims and procedures.

## Results

**Figure 4:**
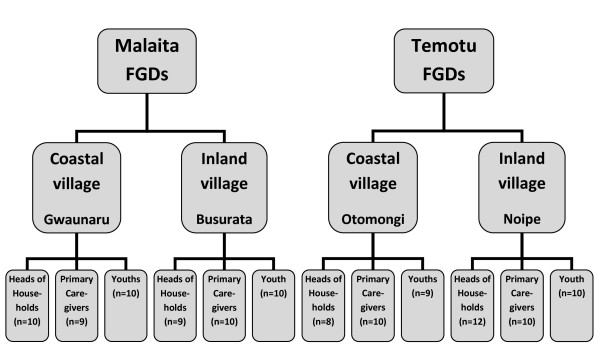
Summary of focus group discussions and number of participants in each.

The number of participants in each FGD ranged from eight to twelve (Figure 4). The age range for household heads and primary caregivers were 25 – 72 years and for youths 16 – 29 years. It was initially intended that FGDs would be carried out with adolescents, but this was interpreted as unmarried young adults by village chiefs during participant recruitment. Therefore, the age range of this 'youth' group is somewhat outside the intended target. Background noise in the rural inland village of Noipe, Temotu resulted in the inaudibility of FGDs with the household heads and primary caregivers in this village. However, information could be retrieved from comprehensive reports written by the research team following these discussions.

### Perceived purpose of bed nets and frequency of use

There appeared to be no gender differentiation regarding the perceived purpose of nets. Protection from mosquito bites was identified by all discussion groups as the main purpose of using a bed net. Many groups went further to say that bed nets protect them from malaria. Participants that lived in coastal villages, where mosquito nuisance, malaria burden and fear of malaria are higher, mostly reported the use of bed nets throughout the year.

'Mosquito net I think is a very good thing especially in our area here and in this village of Gwaunaru, where mosquitoes are too many and I think you might have heard that in this, our area, malaria cases are too high and mosquito net is a very important item in which every family should sleep under it...' (Gwaunaru woman)

'I always sleep under a mosquito net because malaria can kill anyone...die.' (Otomongi man)

Intermittent bed net use was reported by most participants living in areas with low or seasonal mosquito nuisance.

'For me, I know...I am from the seaside (coast) and I know that mosquitoes bite all the time, then I got married to a man from the bush and I came to know that it is a bit different in the bush...because...sometimes we do not feel any mosquitoes biting here, and mosquitoes are not many as at the seaside (coast).' (Busurata woman)

Participants using bed nets intermittently were unable to describe specific months of the year when they were used except to say that it was usually when mosquitoes were present; if someone in the family had malaria; and/or during the cooler months of the year.

'We don't use our nets when there are no mosquitoes around. But we use them when any member of our family sick after testing positive with malaria...but later we will then fold them back and keep them.' (Busurata man)

Other factors affecting bed net use mentioned by participants were the perceived benefit and effectiveness of the nets in preventing mosquito bites and malaria; having sufficient nets for household coverage; knowledge regarding insecticide in the nets and instructions in their use and care; protection from other nuisance insects such as flies, ants, cockroaches and lice; and the difficulty and expense in seeking treatment for malaria. Distribution of an individual's preferred net and having a choice over sizes of nets provided were also mentioned by a few participants as motivators for their increased use.

Children are often prioritized for use of bed nets as many participants reported insufficient net coverage for all members of the household. Reasons for this included; insufficient free distribution of new bed nets for the entire household (due to current bed net distribution policy in SI targeting vulnerable populations such as children and pregnant women at the time of the study); old nets that are torn or were taken away and replaced with new nets by Ministry of Health staff; and insufficient household funds for net replacement.

'...there were not enough nets supplied to fit the whole family.....also there is not enough money to cater for the required number of nets for the family.' (Busurata man)

'(I have) none either, you know the system now, they take them away.' (Gwaunaru youth)

'Those that distribute the nets distribute them only for the children.' (Gwaunaru man)

Participants in Otomongi, a coastal village in Temotu, were alone in reporting sufficient bed net coverage for their households. Bed nets are reportedly used year round in Otomongi due to high mosquito nuisance and fear of malaria.

Finally, it was suggested that those living in houses made of bush materials had a greater need for bed nets than those in semi or permanent houses due to increased mosquito nuisance inside a bush house. As a Busurata woman describes; *'...because the doors in our home are always closed and we also have window screens to prevent the mosquitoes coming in the house...when the mosquitoes bite, they do not come in the house so we do not use the nets that much.'*

### Sleeping patterns

Sleeping arrangements in households were investigated as it was thought that these may play a role in bed net use. It was found that where people sleep varied between households and can depend on the number of bed nets available and the number of rooms or beds there are. Generally, arrangements are similar between Temotu and Malaita Provinces with the exception of older boys (from the age of 14–15 years). In Malaita Province, once boys reach this age they have a lodge built (young man's house) next to the main family home. In Temotu, boys from this age traditionally sleep in the village 'single house,' (a group house, known as *Madei *in Santa Cruz) which is a place shared with older men where custom is taught. In both Provinces, small children usually sleep with their parents or just the mother (and sometimes with aunties, grandmother or a 'house girl'). Older children usually sleep in a separate room from the parents if available, some board at their school if it is some distance from their village and some may visit relatives in other villages.

Discussions in all study villages reported variability in the locations that men sleep. These included with their wife in the main house, in their own room, in the kitchen (which may be outdoors), on the veranda or around a fire. Outdoor sleeping of men was reported in both rural and coastal villages (i.e. despite differences in mosquito nuisance), however, in Otomongi (coastal village in Temotu) greater bed net use was reported due to mosquito nuisance and fear of malaria. The sleeping location can also differ night by night depending on climate and work or evening activities as described by one Noipe youth; *'...some sleep in the kitchen, beside the fire, some sleep in the sitting room and some inside the room...some do not use mosquito nets.'*

### Misconceptions of long-lasting insecticidal nets (LLINs)

Although the specific names of the long lasting insecticidal nets were not generally known, participants were able to descriptively differentiate between them. The two main types of nets used in the villages visited were Olyset^® ^(often referred to as 'the one with the big holes') and PermaNet^® ^(referred to as 'the soft one'). Participants reported that minimal information was provided at the point of distribution of their LLINs especially those received through a nurse aid post (NAP) or provincial hospital. Although the majority of participants knew the new long lasting nets were pre-treated, there was still considerable confusion as to how long the insecticide would last, whether washing would affect it and whether they needed retreatment. The ongoing existence, availability and use of older insecticide-treated nets (ITNs) (that require re-treating every six months) and cotton nets in their village may be contributing to this confusion. A few participants also commented that all bed nets pose a hazard to infants and children and there is a dislike of the side effects.

'What I heard from some people, because since they spray the net, some people are afraid to sleep under it because when they sleep under it and breathe the medicine they cough too much.'

*'Some say that when they sleep under mosquito net they are breathless.... and some say they don't like the smell of it, of the medicine. That's what I heard some people say*.'* (Gwuanaru women)*

Much of the rumour and misunderstanding surrounding LLINs centred on Olyset^® ^bed nets. Many participants perceived Olyset^® ^nets to be ineffective as they believed mosquitoes can penetrate the nets through the larger mesh size and that the insecticide strength diminishes with washing. Table 2 further details some of the comments made by participants during the FGDs.

**Table 2:** Summary of perceptions and misconceptions of long lasting insecticidal bed nets

Province	Quotes
Malaita	*'...when we sleep inside the net and it looks dirty we will wash it...I think that's what makes malaria not stopping...I don't know.' (Gwuanaru woman referring to LLINs in general)*
	*'What I heard from some people is that ... some people are afraid to sleep under it because when they sleep under it and breathe in the medicine they cough too much.' (Gwuanaru woman referring to LLINs in general)*
	*'...some types of mosquitoes are small ones and they can go in through the net...' (Gwuanaru youth referring to Olyset nets)*
	*'...I'm really worried that the medicine on the net may wash out.' (Busurata man referring to LLINs in general)*
	*'I don't wash mine because I fear that the insecticide will be washed out...so sometimes it stays unwashed for the whole year.' (Gwuanaru man referring to LLINs in general)*
	*'When the baby wakes up from sleep and cries it usually rolls on the bed and if we don't hurry to get the baby he/she can become trapped in the net and therefore become breathless. It is a serious situation that can be dangerous...' (Busurata man referring to bed nets in general)*
	*'...they say children should not hold the mosquito nets because if you hold them then eat, you are going to die. If you hold it, you must wash your hands.' (Gwuanaru woman referring to LLINs in general)*
	*'But it is the children, when they sleep under the net they can get sick because it has big holes' (Busurata woman referring to Olyset bed nets)*

Temotu	*'When you take out the mosquito net for use (Olyset net), keep the plastic bag, fold it well and keep it safe, because if its longer than or up to a few months, to return its medicine, you have to fold it well and put it back in the plastic bag.' (Otomongi woman)*
	*'...you wash it and hang it to dry, then you put it in the plastic again because the insecticide is still in the plastic...' (Noipe youth referring to Olyset nets)*
	*'...you wash it, but keep the plastic in a safe place...and after you wash it, you hang it out in a shade place...when you see that the water has dripped off from the net, you put it back inside the plastic...from morning till evening, then you hang it up and you can sleep under it after that.' (Noipe youth referring to Olyset nets)*
	*'For me, though it's dirty I will still keep it until the malaria team comes to retreated it then that is when I will wash it again.' (this Otomongi man reports to have Olyset & PermaNet bed nets in his household)*
	*'Especially when the mosquito is not sucking blood yet and it is still small, it can go through the holes, then when its full of blood it cannot get out, therefore, in the morning you will see a lot in the net...' (Otomongi woman referring to Olyset nets)*
	*'You can get sick with that net (Olyset).' (Noipe youth)*
	*'...I think you can tell them to send us some good nets that are soft and the holes are small so that mosquitoes cannot go in...because this one here (Olyset), even though it is treated, in the morning when you wake, you will see some mosquitoes flying inside the mosquito net and filled with blood...ah, what kind of net is this...' (Noipe youth)*

### Maintenance of bed nets

Participants described considerable variation in the frequency of washing of LLINs. Bed net washing reportedly occurred whenever the nets were dirty; every few weeks; every few months; once a year; and sometimes never. Participants were not aware of the recommendations of how often their nets should be washed. In addition, many participants were able to describe that they needed to place their Olyset^® ^nets in a plastic bag to reactivate the insecticide (after washing), yet they did not appear to fully understand the process, and some believed that the 'medicine' was in the plastic bags.

Some participants were also concerned that the insecticide would come out with washing and others were awaiting the return of the malaria team to retreat their Olyset^® ^or PermaNet^® ^nets (as per the traditional nets that required washing a day or two before retreatment was due). One participant also reported that infrequent washing of bed nets was due to the difficulties of not living in proximity to water.

### Favourable and unfavourable characteristics of LLINs

Some general problems reported with the use of LLINs were that the white coloured nets dirty easily. Darker colours are preferred and larger sizes requested given that the nets are reportedly not wide enough to accommodate the size of the sleeping mats (made of coconut leaves) used in the villages.

'...instruction says when you sleep under it, you tuck the net under the mattress. But we in the village don't have mattress, (mattress can hold the net down while we're sleeping), but we have mats that make it quite hard to do that.' (Otomongi youth)

'...they say to put the net under the mat, but with these mats made of coconut leaves, there sizes are bigger than the nets, so it doesn't stay like this, the thing that can happen is it will break it.' (Otomongi youth)

During the focus group discussions, participants were given the opportunity to examine three different brands of LLINs (Olyset^®^, DuraNet^® ^and PermaNet^® ^nets) and comment on the favourable and unfavourable characteristics of each (see additional file 1: summary of favourable and unfavourable characteristics of LLINs).

#### Bed net acceptability

Overwhelmingly, participants expressed a lack of acceptability for Olyset^® ^bed nets due to its unfavourable characteristics (namely its large mesh size that is perceived to allow small mosquitoes to fly through and its tendency to wrinkle and shorten with use making it difficult to keep securely tucked under sleeping mats). Most participants reported both DuraNet^® ^and PermaNet^® ^to be acceptable. There did not appear to be any significant differences in LLIN acceptability between primary caregivers, household heads and youth groups, nor between sample populations of Malaita and Temotu Provinces. The few participants that did not consider PermaNet^® ^to be acceptable reported the grounds for this as its lack of durability, while others reported that they would use any net provided if there was no other choice, as long as it protected them against malaria. DuraNet^® ^was deemed acceptable by all groups as its characteristics found the middle ground between the unfavourable characteristics of Olyset^® ^(mesh size too large) and PermaNet^® ^(perceived lack of durability). The main criterion for bed net acceptability identified by these FGDs was its perceived effectiveness in preventing mosquito bites and malaria. This was affected by the inherent characteristics of the net such as mesh size and texture. As detailed in Table 2, perceptions and misconceptions regarding LLINs, their insecticides and how to use and care for these nets, also appeared to impact on acceptability.

A lack of affordability or availability of alternative options meant that despite Olyset's^® ^lack of acceptability, participants reported that they would use these nets if these were the only type made available. However, this mostly occurred in areas with (or in times of) higher mosquito nuisance and amongst participants that were aware of the cause of malaria and perceived it as a significant threat to the well-being of themselves and their family.

'...but since I don't have any other type of net I will have to use it (Olyset net), even though the mosquitoes bite me when I use it, I will have to sleep in it.' (Gwaunaru woman)

' If they (the malaria people) brought only one (type of net), we cannot say I would like another one......all that we need is to (be) safe from mosquitoes.' (Busurata man)

Participants from the inland village of Noipe, Temotu where mosquito nuisance is low and seasonal were particularly explicit in reporting that they would not use Olyset LLINs even if it was the only net available.

*'Me too, I will not accept the blue one (Olyset); I like these other two nets (DuraNet & PermaNet).'* (Noipe Youth)

### Bed net preference

There was an overall preference for DuraNet^® ^bed nets because of their strength, and a mesh size that was smaller than Olyset^® ^(which they believe allow mosquitoes to penetrate) but larger than PermaNet^® ^(which limits ventilation). It was acknowledged in many of the discussions that DuraNet^® ^bed nets have not yet been trialled in Solomon Islands and their preference was based on anticipated performance of the net. In Temotu province a preference was expressed for DuraNet^® ^and PermaNet^® ^almost equally, with a strong dislike for Olyset^® ^made known. There did not appear to be any significant differences in bed net preference between primary caregivers, household heads and youth groups.

### Increasing bed net use – participant recommendations

Two key recommendations arose from the communities involved in the study. Firstly, regular awareness programmes were proposed that should be specifically tailored to reach the poor, the isolated and the illiterate/uneducated. It was suggested that these programmes clarify misconceptions and include education on malaria and it's transmission; the personal and economic benefits of reducing malaria; the benefits of bed nets, how they work and how long the insecticide lasts; and instructions on their use and care.

'During the (malaria) programmes before I saw people stay healthy...that is why we always need awareness.' (Otomongi man)

It was further suggested that local people be provided training and incentive to carry out education and awareness activities in more remote areas to increase coverage of the programme.

Wrongful use of bed nets were reported by a few participants during the discussions and it was suggested that both old and new nets are used for fishing and protecting the 'nursery' (cabbages and other small crops). Similar misuses of bed nets were reported by a recent study in Kenya [16]. Participants in the current study therefore proposed that any education programme also address the prevention of wrongful use of bed nets.

Secondly, since a lack of household financial resources was reported as one of the main barriers to bed net ownership and use, it was proposed that nets be provided by the Ministry of Health free-of-charge and in sufficient quantity and sizes to achieve full household coverage. A final issue of interest that was raised by household heads in one village was a distrust of 'malaria people' in their selling of bed nets as noted below:

'I think the nets that were sold to us in the past were meant to be freely distributed to us, but I think it is the malaria people who came to sell the nets to us for their own pocket money...we sometimes have this thought in our minds...because sometimes they said that it cost six dollars, and sometimes they just sell the nets for four dollars.'

'We think that they are making money for themselves.'

## Discussion

### Bed net acceptability

The main criterion for bed net acceptability identified by this study was its effectiveness in preventing mosquito bites and malaria. Both PermaNet^® ^and DuraNet^® ^were deemed acceptable by participants because of their perceived effectiveness. DuraNet^® ^was mostly favoured over PermaNet^® ^due to its durability and increased ventilation, although, this preliminary indication of acceptability and preference for DuraNet^® ^may not translate following use of the net. A cluster-randomized controlled cross-over acceptability and preference trial of Olyset^®^, PermaNet^® ^and DuraNet^® ^nets was, therefore, carried out following this qualitative enquiry and will be reported separately. This study did not identify gender differences in bed net acceptability or preference.

The general experience of participants having used Olyset^® ^nets was that mosquitoes were able to penetrate the net and did not afford them full protection. For this reason, Olyset^® ^nets were generally not seen as acceptable. A study of five malaria endemic countries in South America also found that the acceptability of malaria interventions is related to people's perception of an immediate protective effect against mosquitoes [17].

Olyset's^® ^perceived inability to provide an acceptable level of protection may be caused by a number of factors. Firstly, although participants reported that the mosquitoes are able to pass through the mesh holes, it is more likely that they enter under a net that has become untucked from the sleeping mat. Some participants explained that the sleeping mats used in the villages were often wider than the nets provided and hence Olyset's^® ^tendency to wrinkle and shorten prevents the net from being securely tucked beneath the mat.

Secondly, the surface dose of permethrin should have a repellent effect on the mosquitoes. However, this surface dose can be affected by heat, dust, dirt, light and wind [18]. In addition, laboratory tests have shown that 76% of surface permethrin on Olyset^® ^nets are lost through one wash with soap, however, 82% is regained by being exposed to strong sunlight, in accordance with manufacturers washing and regeneration instructions [19]. A cohort study carried out in Western Kenya similarly found a significant correlation between inadequate maintenance of Olyset nets and failure of its biological efficacy [20]. A recent study in Sri Lanka revealed varying information given at the time of bed net distribution regarding their maintenance and use, and little dissemination of these instructions within the family [21]. The considerable confusion around Olyset's^® ^maintenance and washing instructions reported by participants in this study may be contributing to its reduced effectiveness in repelling mosquitoes, preventing bites and malaria and thereby reducing its acceptability in Solomon Islands. In contrast, a field effectiveness study of PermaNet^® ^bed nets showed that biological efficacy against mosquitoes was similar between washed and unwashed nets up to 18 washes [22].

### Compliance

Mosquito nuisance and perceived threat of malaria were the main determinants of bed net use in those participants who owned a net. Knowledge of malaria and the means to prevent it were not sufficient to guarantee compliance with bed net use. Bed nets were described as being used primarily when there is high mosquito nuisance or if participants perceived malaria as a significant threat to the health of themselves or their families. Other studies from South America, Africa, Malaysia, Papua New Guinea and Solomon Islands report similar findings and describe factors including mosquito susceptibility to the insecticide, high mosquito densities when people go to bed and high malaria transmission as important for compliance and the success of bed net programmes [9,17,23-25].

A commonly reported reason for non-use of bed nets was a lack of ownership of a net. Participants attributed this to inadequate household coverage of bed nets during past distribution campaigns, a lack of durability of nets distributed (especially PermaNet^®^), and a lack of household financial resources to purchase additional nets. However, Otomongi, a coastal village in Temotu reported good household coverage and use of bed nets. Participants in this village also reported higher mosquito nuisance and malaria transmission. It is unclear whether good household coverage of bed nets in this village is due to recent net distribution in the area or a result of households prioritizing the purchase of bed nets in their budgets as a consequence of mosquito nuisance and perceived threat of malaria. This will need further investigation.

Bed net acceptability and preference appeared to play a varying role in compliance with bed net use in the villages involved in this study. Participants in areas of high and year round mosquito nuisance and perceived threat of malaria often reported the use of bed nets regardless of their unfavourable characteristics due to the lack of availability or affordability of alternative options. Those in areas of low and/or seasonal mosquito nuisance (who perceived threat of malaria as lower) were more likely to describe the unfavourable characteristics of bed nets as reasons for their intermittent compliance or non-use. Therefore, providing nets that are acceptable and/or preferable will be more important for improving compliance in areas of low or seasonal mosquito nuisance and malaria transmission.

Gender differences were not noted regarding perceived purpose of using bed nets, however, there appeared to be differences in compliance with bed net use between men and women. Lifestyle factors, such as the changeable sleeping arrangements of men (and sometimes older male children), played a significant role in compliance with bed net use. Climate, work and evening social activities impact on where men choose to sleep on any particular evening. In contrast, the relative stable sleeping arrangements of women, their practice of sleeping with their young children and because bed nets are reportedly prioritized for children suggests that it is more likely that compliance is higher among women, particularly in areas of low or seasonal mosquito nuisance.

### Implications for malaria elimination

Issues of acceptability, preference, poor compliance, and insufficient or incorrect maintenance of nets pose significant challenges for malaria control and elimination in Solomon Islands and elsewhere. Mass distribution of acceptable or preferred bed nets accompanied with education for behaviour change may initially improve compliance with their use. Standardized verbal and written education should include information on the insecticide, its expected longevity, potential side effects (and advice should these occur), and instructions on maintenance and washing of the nets. This information will require regular reinforcing and, as suggested by the study participants, may be best provided by trained community volunteers able to deliver regular and locally appropriate instruction. Approaches developed with communities are required in order to maximize message delivery, comprehension and retention; such as the Japan International Cooperation Agency (JICA) funded project in Guadalcanal that uses pictorial flipcharts in community-based health information workshops.

The elimination programme in Solomon Islands will need to address the issue of changeable sleeping practices of men, which increase their vulnerability to malaria. Education will need to be tailored to engage men in participating in personal protection measures. The promotion of complementary protection methods such as the application of repellents (soaps or lotions) or burning mosquito coils could accompany bed net distribution to account for lifestyle factors that expose the population to malaria prior to retiring to bed.

In areas of low and/or variable mosquito nuisance, education will need to emphasize that the risk of malaria is not diminished when mosquito numbers are low. In addition, it should be highlighted that LLINs will reduce the risk of malaria, but not eliminate it. This may reduce the disillusionment that can occur when bed nets do not meet the expectation of preventing malaria and may help to increase their acceptability. Monitoring and evaluation to ensure guidelines are met during a national bed net distribution and education/awareness campaign will be essential to prevent inconsistencies that may lead to mistrust of health staff which can impact on net acceptability, ownership and use.

As elimination successfully proceeds and transmission and perceived threat of malaria diminishes, it is likely to become more difficult to maintain compliance, particularly in areas with low or seasonal mosquito nuisance. Compliance has previously been described as a function of perceived risk of a disease and the belief that it can be prevented and controlled [26]. However, a study in Malawi showed no evidence that perception of malaria risk directly influenced compliance with control interventions [27]. Rather, the author suggests that preventative behaviour may be determined by other social mechanisms, and that interventions should be structured to utilize such mechanisms [27]. Therefore, sustainable participation and compliance with vector control methods (such as bed net use and IRS) may be more likely achieved through community consultation and engagement; and the facilitation of a process that enables and empowers communities in the development of their own solutions. This community-directed approach underpins the Solomon Islands Government strategy for intensified malaria control and elimination and will be vital for maintaining compliance despite an absence of perceived immediate or direct threat of malaria.

### Study limitations

This study was conducted to explore issues around bed net acceptability, preference and use, as well as to inform a subsequent cluster-randomized controlled cross-over trial. As with the nature of qualitative research, the results are limited in their ability to be generalized to the wider population. However, the finding that there was no significant difference in acceptability of LLINs between such geographically remote study samples would suggest that similar responses may be found elsewhere in SI. Another limitation of the study may be the potential loss of nuances that can occur through the direct transcription and translation from Pidgin to English by the local research officers. The availability for recruitment of only male field researchers may have also had an impact on the ability to identify gender differences in bed net acceptability or preference. Finally, the authors recognize that responses on ownership and frequency of bed net use may be an overestimation as these are subject to the potential bias of self-reporting.

## Conclusion

This study has identified the characteristics of bed nets that affect their acceptability and preference and has outlined some of the potential barriers affecting compliance with their use in Solomon Islands. It has been used to inform a subsequent randomized controlled trial to determine the degree of acceptability and preference of Olyset^®^, PermaNet^® ^and DuraNet^® ^bed nets (described elsewhere). Further research is required to understand health priorities of communities; their knowledge, perceptions and behaviours relating to malaria; motivational drivers for participation in malaria elimination; and an investigation into the key stakeholders that could support the programme. In particular, a participatory action-orientated approach will assist in building partnerships with communities and engage them in the process of local decision-making and implementation. This will be integral to the success and sustainability of the elimination programme in Solomon Islands.

## Competing interests

Several Olyset^® ^and DuraNet^® ^LLIN samples were supplied by their respective distributors free of charge for use during the focus group discussions to allow participants to inspect each net for their favourable and unfavourable characteristics. PermaNet samples were already available in-country. However, the authors declare that they have no competing interests.

## Authors' contributions

All authors participated in the conception of study design. Training of field researchers in qualitative methods and logistics was carried out by LF and JA. The field research activities were supported by AB, LB, BA, JA, AV & LF. Data analysis and manuscript drafting was carried out by JA with support and contributions from all authors.

## Supplementary Material

Additional file 1 **Summary of favourable and unfavourable characteristics of long lasting insecticidal bed nets identified in the focus group discussions**. Click here for file
