# Supplementary material for: A qualitative study on the acceptability and preference of three types of long-lasting insecticide-treated bed nets in Solomon Islands: implications for malaria elimination
Source: Malar J. 2009 Jun 4;8:119. doi: 10.1186/1475-2875-8-119 (PMC2699345; doi:10.1186/1475-2875-8-119)
Supplement: Additional file 1 — Summary of favourable and unfavourable characteristics of long lasting insecticidal bed nets identified in the focus group discussions. [file 1475-2875-8-119-S1.pdf]

Additional file 1: Summary of favourable and unfavourable characteristics of long lasting insecticidal bed nets identified in the focus group discussions.

LLIN	Favourable Characteristics	Unfavourable Characteristics	Quotes
**Olyset**	- Strong and durable<br>- Increased ventilation provides comfort with sleeping<br>- Claimed longevity of insecticide<br>- Easy to hang as metal rings at corners allows wooden stake to be placed along 2 margins of net upon which ropes are tied. This makes nets easier to hang as it provides a 2- rather than 4-rope attachment system, which is more practical in small village houses.	-Large mesh size is perceived to allow small mosquitoes to fly through<br>-Wrinkles and shortens with use and no longer stays tucked under sleeping mats during the night<br>-Net melts and holes are created when in close proximity to heat from lamps<br>-Rough and difficult to wash<br>-Babies get cold during the night and become sick due to increased ventilation	*'...the only problem with it (Olyset net) is the ends can wrinkle and fold up and the mosquitoes can fly in...that is not good...and sometimes the big holes...little mosquitoes can fly in through...and sand flies too...that is the disadvantage of the net.' (Busurata woman)*<br>*'...since in the village we have kerosene lamp, then if you want to study or do anything, they (the mosquitoes) can come and disturb you so you have to take your lamp inside the mosquito net, it's not good that if you put the lamp close to it (Olyset net)...it will melt.' (Otomongi youth)*<br>*'That blue mosquito net (Olyset) cannot dirty easily. It can also last long. It could only get dirty if children are careless. Even though the dust/dirt from the roof falls on it, it can still looks as when it was still new. So that is why I also like that one....the other good thing about it too is the fresh air.' (Busurata man)*<br>*'Ok, in my view...the holes are big and I think that mosquitoes can fly in. If the holes are smaller, I think it is a nice one because it is strong.' (Busurata youth)*
**PermaNet**	- Soft and flexible which allows net to lie flat to the ground (no wrinkling)<br>- Mesh size small which prevents mosquito penetration during the night<br>- Sufficiently wide to allow comfortable sleep	-Net breaks easily by restless sleeping or by children<br>-Claimed longevity of insecticide isn't as long as Olyset and DuraNet<br>-Hot to sleep under due to smaller mesh size<br>-Gets dirty easily because small mesh size traps dust and dirt that falls from the roof of village houses<br>-Colour fades easily<br>-Height of net is too short	*'In my observation; one thing is that, little children like to play inside the nets.... Sometimes they play inside and it breaks.' (Gwuanaru woman)*<br>*'If it has a little break, the net will start to break bigger from that hole. When the children roll, they will break it more.' (Otomongi woman)*<br>*'We use PermaNet in my home but when I take care of it, because I am an adult, it lasted a long time.' (Busurata woman)*<br>*'(PermaNet) is a bit hot. During hot seasons it's too hot.' (Busurata man)*<br>*'The yellow one here I also like too. When I get cold and sleep in side, it keeps me warm.' (Busurata man)*<br>*'PermaNet is a good one but it can be hot as well…and if there are two or three people sleeping under, and they move in and out of it, it will be hot inside the net…' (Gwuanaru man)*<br>*'...it is a nice net…it is cool, soft and it does not squeeze up and make spaces that mosquitoes can go in; when it lays down it lays down straight on the mat (when tucked).' (Noipe youth)*<br>*'It is not strong; but it is soft and that is nice. It falls straight down following the bed…no problem with it…' (Busurata woman)*

**DuraNet**	- Strong and of good quality<br>- Mesh size small enough to prevent mosquitoes and sandflies flying through and large enough to allow good ventilation and a pleasant night's sleep<br>- Claimed longevity of insecticide important for those living in isolated areas where 'malaria people' don't visit often	-May share some of Olyset's disadvantages as it is made of the same material<br>-Net never been trialled in SI so participants found it difficult to identify unfavourable characteristics	*'...its holes are not too big or too small...' (Noipe youth)*<br>*'It's good because its holes are small, so mosquitos cannot go in and even the flies.' (Otomongi youth)*<br>*'It is strong and does not break easily like the other one (PermaNet)...' (Busurata woman)*<br>*'This one looks like when one sleeps under it, it can be cool inside; it is not like this one (PermaNet), where it is hot...' (Gwuanaru man)*<br>*'Because it is strong…it suits those of us living in the rural areas…' (Gwuanaru youth)*<br>*'Although we haven't used it, it will be the same with that other one (Olyset). If we use it for a long time it might also have the problems of that one.' (Otomongi man)*<br>*'I think its problems will be the same as the blue one (Olyset) like, wrinkle, shrink.' (Otomongi youth)*<br>*'Yeah, it is strong…but I don't like it..…that one (PermaNet) lies flat...so (DuraNet's) fault is, I want it to be soft as PermaNet.' (Noipe youth)*
